# Predation risk shapes among- and within-individual variation in behavior in poison frog tadpoles

**DOI:** 10.1093/beheco/arag041

**Published:** 2026-04-20

**Authors:** Josie McPherson, Julien G A Martin, Lauriane Bégué, Noëlle Tschirren, Patrick Walsh, Eva Ringler

**Affiliations:** Institute of Ecology and Evolution, University of Edinburgh, Edinburgh EH9 3FL, United Kingdom; Department of Biology, University of Ottawa, Ottawa, Canada K1N 6N5; Division of Behavioral Ecology, Institute of Ecology and Evolution, University of Bern, Bern 3032, Swizterland; Division of Behavioral Ecology, Institute of Ecology and Evolution, University of Bern, Bern 3032, Swizterland; Institute of Ecology and Evolution, University of Edinburgh, Edinburgh EH9 3FL, United Kingdom; Division of Behavioral Ecology, Institute of Ecology and Evolution, University of Bern, Bern 3032, Swizterland

**Keywords:** animal personality, behavioral plasticity, predictability, predation risk, *Allobates femoralis*, tadpoles, behavioral syndromes, consistent individual behavioral variance, DHGLM

## Abstract

Environmental conditions during early development can considerably shape individual behavior, and laboratory studies suggest that predator presence is an important factor influencing both within- and among-individual behavioral variation. To test this under natural conditions, we studied neotropical poison frog (*Allobates femoralis*) tadpoles from pools with and without dragonfly larvae (tadpole predators) under naturally varying environmental conditions. We measured 2 behavioral traits, distance moved in a novel environment and time to emerge from a shelter, across 4 repeated trials in predator-exposed (*n* = 48) and predator-naïve (*n* = 169) tadpoles. Predator-exposed tadpoles moved less, emerged later in their first trial and were more unpredictable in emergence time than predator-naïve individuals. Moreover, among-individual variation in both behaviors was higher in predator-exposed tadpoles, although behavioral repeatability did not differ significantly between predator-exposed and predator-naïve groups. Predator-naïve individuals exhibited behavioral and predictability syndromes, individuals that moved more also emerged sooner, and individuals more predictable in 1 behavior were more predictable in the other. Despite only being significant in predator-naïve individuals, these correlations did not differ between predator-exposed and predator-naïve groups. A personality–plasticity association occurred only in predator-exposed tadpoles: individuals that initially moved less showed less behavioral change across trials. These findings demonstrate that even in naturally variable environments, predation risk shapes behavioral variation at multiple levels, thereby contributing to the emergence and maintenance of “animal personality”.

## Teaser

Growing up around predators changes how tadpoles behave. Tadpoles raised with predators (dragonfly larvae) swam less in unfamiliar environments and left shelters both later and more erratically than those raised without predators. Predator-exposed tadpoles also showed more behavioral differences from one another than tadpoles without predator exposure. This suggests that early experiences play an important role in creating behavioral differences among individuals.

## Introduction

Animal behavior can vary dramatically among-individuals in the same population. For example, in female brown bears, *Ursus arctos*, individuals differed in their diel activity patterns, with some bears being consistently more diurnal while others were more nocturnal; 61% of variation in this behavior was due to individual differences (Hertel et al. 2019). This repeatable among-individual behavioral variation, often termed “animal personality” (Dall et al. 2004; Sih et al. 2004a, 2004b; Réale et al. 2007) is now recognized as a key feature of many animal populations with important implications for ecology and evolution (reviewed in Laskowski et al. 2022).

Individual behavioral variation can be partitioned into three key components that capture different aspects of how animals behave (Cleasby et al. 2015; O’Dea et al. 2022). Personality differences are consistent among-individual differences in behavioral expression, for example, that some individuals are consistently more exploratory than others. Plasticity describes how individuals change their behavior in response to environmental variation (Martin and Réale 2008; Dingemanse et al. 2010; Dingemanse and Wolf 2013; Stamps 2016), for example, in marine gastropod *Littoraria irrorata*, an individual's boldness increased with increasing temperature (Cornwell et al. 2019). Predictability measures how consistent an individual's behavior remains after accounting for environmental effects (Cleasby et al. 2015; Westneat et al. 2015). These components are not mutually exclusive. Personality is typically inferred when among-individual variation exceeds within-individual variation, resulting in repeatable behavioral differences. The same logic applies to plasticity and predictability: repeated measures can reveal consistent among-individual differences in individuals' degree of plasticity or predictability. Studying these components jointly is essential, as they can have distinct causes and evolutionary consequences and may otherwise confound one another (O’Dea et al. 2022; Mitchell et al. 2024). Variation in personality, plasticity, and predictability is increasingly documented across taxa, with early-life experience playing an important role in shaping these traits (Rödel and Monclús 2011; de Jong et al. 2022; Beyts et al. 2023).

Predation represents a strong selective pressure on prey and may be particularly important for shaping individual behavioral variation (Bell and Sih 2007; Dingemanse et al. 2007; Kortet et al. 2010; Scherer et al. 2024). Predators can exert these selective pressures through 2 related mechanisms: consumptive effects, which directly remove certain behavioral types from the population, and nonconsumptive effects, which induce behavioral changes in response to predation risk (Lima 1998; Abbey-Lee and Dingemanse 2019; Dingemanse et al. 2020). Predation risk during early life could affect among-individual variation in opposing ways. On one hand, predation might reduce among-individual variation by favoring a single “optimal” antipredator response, homogenizing behavior within populations (López and Martín 2015; Castellano and Friard 2021), however there is little evidence for this empirically (but see Scherer et al. 2024). Alternatively, individuals may differ consistently in their antipredator responses; in such cases, higher predation risk could amplify among-individual variation rather than reduce it (Castellano and Friard 2021; Scherer et al. 2024). This has been seen in a number of species (Toscano et al. 2014; Ehlman et al. 2019; Tariel et al. 2020; Mitchell et al. 2024).

Predation risk may also shape within-individual variation. For instance, reduced predictability could serve as an antipredator strategy, with less predictable individuals being harder for predators to track and capture (Domenici et al. 2009; Briffa 2013). Similarly, predation risk might reduce plasticity to repeated testing because the costs of inappropriate habituation (decrease in response to repeated stimuli) may be higher when predators threaten survival (Brown et al. 2013; Mitchell et al. 2024). Since repeatability increases with either greater among-individual variation or reduced within-individual variation (Bell et al. 2009), predation's effects on these components will likely alter behavioral repeatability and thus could be key in explaining the existence and maintenance of animal personality.

Beyond affecting personality, plasticity and predictability of behavioral traits separately, predation risk may shape correlations among behaviors. Behavioral syndromes are consistent rank-order differences in individual behavior across time and/or contexts (Bell 2007). In sticklebacks (*Gasterosteus aculeatus*), behavioral syndromes can be predation-dependent, emerging only when predators are present (Bell 2005; Bell and Sih 2007). The presence of predators can influence behavioral correlations, through both selective removal of individuals with particular behavioral correlations and by causing prey to plastically adjust their behavior (Bell and Sih 2007). By the same mechanism, predation could influence plasticity and predictability syndromes (correlations between plasticities or predictabilities of different behaviors; (O’Dea et al. 2022; Sheehy and Laskowski 2023)) and within-behavior correlations (relationships between personality, plasticity, and predictability within a single behavior (O’Dea et al. 2022). For example, bolder personality types may experience greater predation threat. Therefore, they may benefit more from unpredictable behavior as an antipredator strategy, potentially strengthening the correlation between personality and predictability in environments with higher predation pressure (Highcock and Carter 2014).

Anuran tadpoles are known to show strong population behavioral responses to developing alongside predators, including reduced activity (Anholt and Werner 1995; Relyea 2001; Gazzola et al. 2017; Hossie et al. 2017) and increased hiding (Van Buskirk 2001; Gazzola et al. 2017). Recent laboratory work has shown that predator presence during development affects within- and among-individual behavioral variation (Urszán et al. 2015, 2018; Castellano and Friard 2021; Płaskonka et al. 2024). However, laboratory and field approaches each involve methodological trade-offs. Field studies of individual behavior can be constrained by observational limitations and confounded by environmental variables, whereas laboratory studies offer experimental control at the cost of ecological realism. These differences can lead to divergent results, with laboratory and field studies of individual behavioral variation often yielding contrasting patterns (Bell et al. 2009; Moiron et al. 2020). Field studies are therefore essential for evaluating whether these laboratory findings hold under natural conditions.

Here, we examine how predation risk during early life shapes within- and among-individual variation in behaviors in a free-ranging population of Neotropical poison frog tadpoles (*Allobates femoralis*). We compared tadpoles that developed in pools with and without dragonfly larvae predators, measuring both the distance moved in a novel environment and time to emerge from a shelter. We simultaneously examined how initial behavior (behavior at first trial before responding to repeated testing), plasticity to repeated testing (change in behavior across repeated trials), predictability (residual within-individual variation), as well as among-individual variation in behavior, plasticity and predictability and their correlations within and between traits, differed between predator-exposed and predator-naïve tadpoles.

We predicted that, compared with predator-naïve individuals, predator-exposed tadpoles would show: (i) reduced distance moved in a novel environment and increased shelter emergence times as antipredator responses; (ii) reduced plasticity to repeated testing (due to higher costs of inappropriate habituation) and decreased predictability (as an antipredator strategy making individuals harder to track and catch); (iii) increased among-individual variance in behavior, thereby increasing behavioral repeatability; and (iv) stronger behavioral correlations, both within behaviors (between personality, plasticity, and predictability) and among behaviors (behavioral, plasticity and predictablity syndromes).

## Methods

### Study species

*Allobates femoralis* is a Neotropical poison frog (Aromobatidae, sensu AmphibiaWeb 2025), primarily found in the tropical lowland forests of the Amazon basin and Guiana shield. They are diurnal frogs with a highly promiscuous mating system (Montanarin et al. 2011; Ursprung et al. 2011; Stückler et al. 2019). During the reproductive season, males are highly territorial and use a prominent advertisement call to repel male competitors and attract females (Ringler et al. 2011). Females lay clutches in the leaf litter inside the male's territory and after 15 to 21 d of larval development, males transport the newly hatched tadpoles to water bodies outside their territory (Ringler et al. 2015, 2018; Beck et al. 2017). They distribute tadpoles from the same and successive clutches across multiple water bodies (Erich et al. 2015). Adult males vary in their level of aggression (Chaloupka et al. 2022; Peignier et al. 2022), and both males and females display consistent individual differences in their distance moved in a novel environment and shelter emergence (Peignier et al. 2022). In a laboratory setting, tadpoles have been shown to differ in their distance moved in a novel environment and time taken to emerge from a shelter, and these differences are sustained over metamorphosis (Bégué et al. 2024).

### Study population

**Figure 1:**
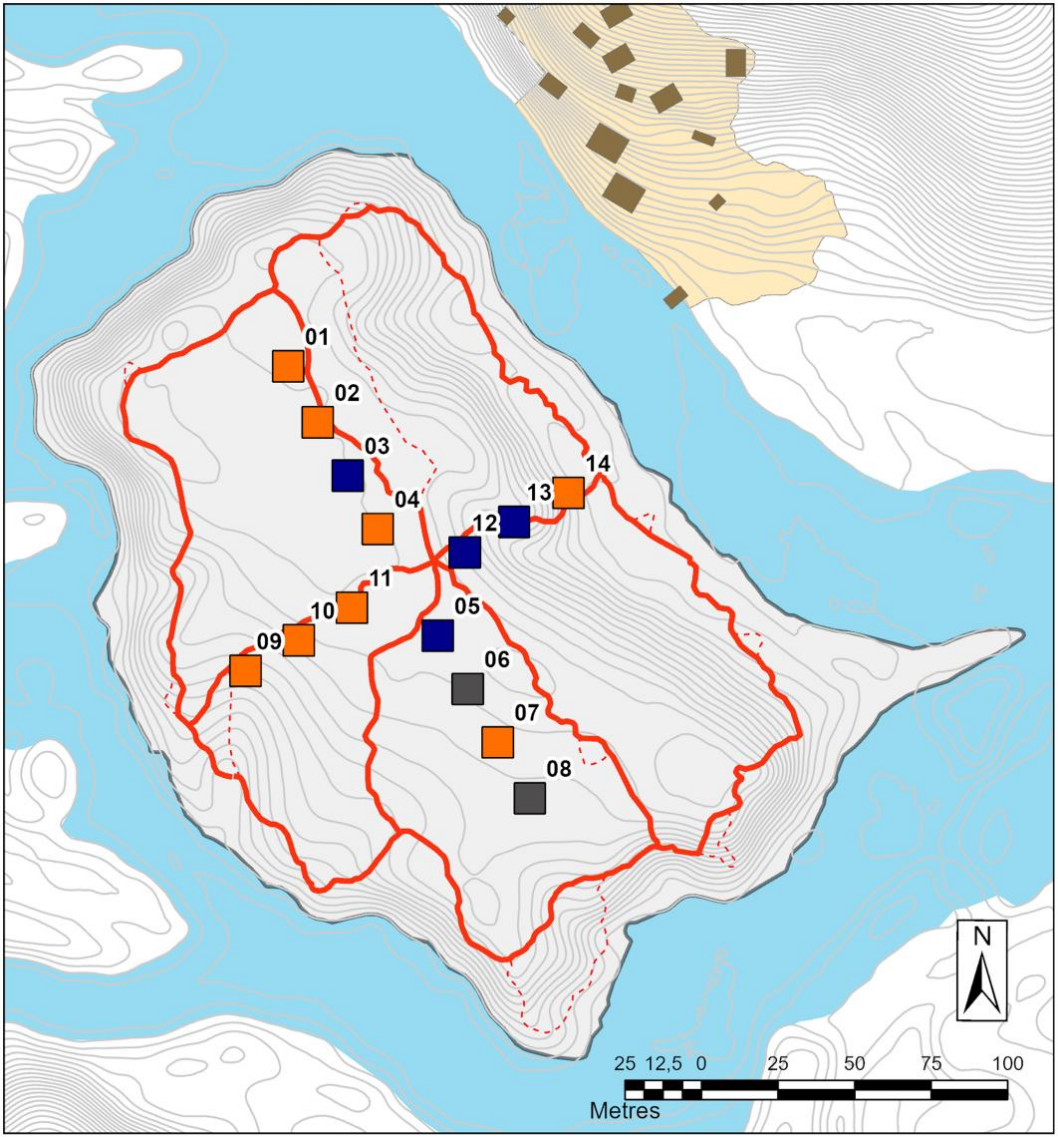
Map of the study area: The pools are represented by numbered, color-coded squares (not to scale). Orange squares are pools with predators (numbered 01, 02, 07, 09-11, 14), blue squares are pools without predators (03, 05, 12, 13) and gray squares (06, 08) are pools without tadpoles. The thin gray lines represent the 50 cm elevation isoclines, the red line represents paths around the island, the light blue area represents the river Arataye, and the yellow area is the “Saut Pararé” field camp.

This study was conducted in a population of *A. femoralis* on a lowland rainforest river island of approximately 5 ha. The island is situated in the river Arataye in the “Les Nouragues” nature reserve in French Guiana (4°02′N, 52°41′W; Ringler et al. 2016; Fig. 1), near the “Saut Pararé” field camp of the CNRS Nouragues Ecological Research Station. The population was introduced in 2012, and in the period of this study the adult population size was 23 adults (for detailed population history, see Ringler et al. 2015).

A cross-shaped array of 14 artificial pools (35 × 35 × 15 cm; Volume: ∼15 L; Fig. 1) was established on the island, where pools within the array are used by the male frogs for tadpole deposition. Pools feature natural variation in a number of environmental factors, such as quantity of leaf litter, amount of shade, and biotic community. Importantly, some pools naturally contain dragonfly larvae and others do not. Dragonfly larvae are a natural aquatic predator of *A. femoralis* tadpoles (Erich et al. 2015; Szabo et al. 2021). As other known aquatic predators such as *Phyllobates tinctorius* tadpoles are absent from the island, dragonfly larvae are the only predator exerting consistent long-term pressure on tadpoles within the pools (Erich et al. 2015; Szabo et al. 2021). While terrestrial predators may occasionally consume tadpoles, such predation represents sporadic events that are unlikely to vary systematically among pools.

### Study design

To investigate how early life predator exposure affected tadpole behavior in this free-ranging population, we originally chose not to manipulate the number of tadpoles or dragonfly larvae in the pools. Two pools did not contain tadpoles during the study's duration and therefore were not included. Of the remaining 12 pools, 8 contained predatory dragonfly larvae of the family Aeshnidae (1.75 ± 0.89 predators) and 4 did not (Fig. 1). After the first batch of behavioral assays, we discovered that tadpole survival was low in pools containing multiple predators. To maintain natural predation pressure while preventing complete tadpole loss, we standardized pools with multiple predators to contain exactly 1 dragonfly larva. Since no tadpoles were present during predator removal, all tadpoles in our dataset experienced consistent predator environments throughout development. The final dataset included 48 predator-exposed tadpoles (6.50 ± 9.38 tadpoles per pool) and 169 predator-naïve tadpoles (54.25 ± 30.84 tadpoles per pool).

The experiment was conducted in 5 consecutive experimental batches between 18 02 2024 and 11 03 2024, with 45 tadpoles tested per batch. To select the 45 tadpoles, pools were selected randomly using a random number generator. In each chosen pool, all tadpoles were counted and the presence/absence of dragonfly larvae recorded. All tadpoles were then removed from the pool and placed into a shallow bucket for the selection process (approximately 10 min). If a pool contained fewer than 9 tadpoles, we included all individuals; if it contained 9 or more, we haphazardly selected 9 tadpoles. Sampling continued until the target of 45 tadpoles was reached. In the final batch, it was not possible to get 45 tadpoles by taking up to 9 from different pools, so 12 tadpoles (rather than 9) were collected from the 2 pools with unsampled tadpoles to complete the count. Overall, 83.33% of the island tadpole population during that time was included in the experiment, with the remaining 16.67% being tadpoles left in these 2 pools at the end of data collection.

To identify individual tadpoles across repeated trials, each selected tadpole was placed in an isolation tube (50 mL falcon tubes; 30 mm diameter, 115 mm height) within their pools. The tubes had mesh bottoms to allow the transfer of aquatic cues and nutrients. To avoid resampling individuals, we did not return tadpoles to their original pool after sampling. Instead, tested individuals were transferred to 5L buckets (filled with rainwater and leaf litter). These buckets were suspended 1 m above ground which prevented new tadpole deposition by males, while allowing tested individuals to continue development. At the end of the experiment, all tested tadpoles were returned to their original pools.

Temperature (25°) and pH (under 6.4), as determined at the lower limit of test strips (AquaActiv) did not differ among pools. Therefore, these parameters were not included in further analyses.

### Behavioral assays

Each tadpole was tested in 2 behavioral assays daily over 4 consecutive days: a novel environment assay and a shelter emergence assay. This resulted in 4 trials of each assay per individual. These assays were chosen because they have been shown to reveal consistent individual behavioral differences in *A. femoralis* tadpoles in a lab setting (Bégué et al. 2024). At least a 1-h gap (mean: 80.24 ± 4.81 min) was maintained between each tadpole's novel environment and shelter emergence assay to minimize carry-over effects. In the first 2 trials, the novel environment assay was conducted first, while in the subsequent 2 trials, the shelter emergence assay was performed first. In both assays, the focal tadpole was placed in a 9 cm diameter Petri dish filled with 50 mL of water (from a well that supplies the Saut Pararé camp). Different Petri dishes were used for the novel environment and the shelter emergence assays, and dishes were cleaned after each use.

A total of 197 tadpoles completed all 8 assays (4 trials × 2 behaviors), while 20 tadpoles completed fewer trials due to metamorphosis (*n* = 4) or mortality (*n* = 16). All individuals were included in analyses as the statistical approach accommodates unbalanced designs (Lee and Nelder 2006).

Tadpole size (snout–vent length) was measured to 0.01 mm using ImageJ (Schneider et al. 2012) from digital photographs taken alongside millimeter scale paper at the end of each experimental batch or the last available assay an individual completed. Size did not differ between predator-exposed and predator-naïve tadpoles (unpaired *t*-test, *t* = −0.23, df = 57.3, *P* = 0.82).

### Novel environment assay

To assess the exploratory behavior of tadpoles, we recorded their movement in a novel environment. Each tadpole was placed individually in a Petri dish, which was then covered with a black plastic sheet for 5 min to allow acclimation and minimize capturing behavioral disruption from handling. Following this acclimation period, the cover was removed, and the tadpole's behavior was filmed for 10 min using a video camera (GoPro Hero10). To increase processing speed, videos were resized to 640 × 360 pixels and reduced to 5 frames per second using the command-line tool ffmpeg (Tomar 2006). We measured total distance moved (in pixels) during the 10-min period using a custom Python script (see Data Availability, based on Beyts 2022). Total distance moved in 10 min was chosen based on previous studies in adult and tadpole *A. femoralis* (Peignier et al. 2022; Bégué et al. 2024) where distance traveled best captured individual differences in exploratory behavior.

### Shelter emergence assay

Here, each tadpole was placed in a Petri dish with an opaque lid cut into 2 halves and allowed to acclimate for 5 min with the lid on. After acclimation, both lid halves were briefly lifted to determine which half of the dish the tadpole occupied. The lid was then replaced over the side the tadpole occupied, creating a “sheltered” area, while the opposite half was left uncovered to form an “exposed” zone. Tadpole behavior was recorded for 10 min and analyzed using BORIS software (Friard and Gamba 2016). We measured the time from the removal of half the lid until first emergence from the covered area. Tadpoles that did not emerge within the time period were assigned a censored value of 600 s.

### Ethics

This study was approved by the scientific committee of the “Nouragues Ecological Research Station”. The behavioral experiments were conducted in accordance with current French and EU law, in accordance with the Study of Animal Behavior (ASAB) guidelines.

### Statistical analysis

We used a multivariate double hierarchical generalized linear model (DHGLM) to simultaneously analyze the among- and within-individual (co-)variation at the mean (initial behavior and temporal plasticity) and residual variation levels (predictability) (Lee and Nelder 2006; Cleasby et al 2015). A DHGLM can be described as a combination of 2 mixed models with the mean model that fits the variation in the mean (similar to a standard mixed model), and a dispersion part that models the heteroskedasticity in the residual variance. The DHGLMs were fitted using the brms package (Bürkner 2017) in R version 4.3.3 (R Core Team 2024) that fits Bayesian multilevel models using Stan software (Stan Development Team 2020).

The DHGLMs were fitted using 4 Markov chains, each including 8,000 iterations, 3,000 burn-in iterations, and a thinning interval of 10. We used weakly informative priors for all parameters. For fixed effects we used a Gaussian distribution with mean 0 and variance of 1. For random effects, we used half student distribution with (*t*(3, 0, 2.8)) and a LKJ distribution priors for the correlations (lkj(2)). For all parameters, autocorrelation was below 0.1, all chains had converged (−R^ below 1.1) and effective sample size was large (Gelman et al. 2013). Posterior predictive checks showed an adequate fit of the model. For all parameters we reported the posterior mean and the 95% highest posterior density interval (HPDI).

To estimate among-individual variation in intercept (how individuals behave on their first trial, before any experience with repeated testing, referred to as “initial behavior”), slope (plasticity to repeated testing) and residual variation (predictability) as well as their within- and between-traits correlations, we fitted a multivariate DHGLM with the traits measured in the 2 different assays as response variables: distance moved in a novel environment (in the 10 min period of the assay) and time to emerge (time in seconds taken before first emergence from the shelter). Individual identity was fitted as a random effect in both the mean and dispersion part of the model for both traits.

Since distance moved in a novel environment is a strictly positive variable, it was log-transformed prior to analysis to address potential constraints in the mean-variance relationship. Additionally, because the time until tadpole first emerged was capped at 600 s, it was modeled using a censored Gaussian distribution with bounds from 0 to 600 s. Both response variables were scaled and mean-centered.

#### Effect of predation at the group level

In the mean part of the model, to assess the effect of predation presence on behavior at the group level, we included a fixed effect of predation presence fitted as a binary variable of presence versus absence. To estimate how tadpoles changed in their distance moved from trial 1 to 4 (plasticity to repeated testing), we included a fixed effect of trial number, fitted as a continuous covariate with the first trial coded as zero. To be able to look at how predation exposure affected plasticity to repeated testing, we included an interaction term between trial and predation presence.

The dispersion part of the model also contained a fixed effect of predation presence (binary presence versus absence) which allowed us to investigate how predation presence affected predictability at the group level.

#### Effect of predation on the magnitude of among-individual variance and correlations

The among-individual variance and covariances were estimated separately for predator-exposed and predator-naïve individuals. To evaluate the difference between predator-exposed and predator-naïve groups, we computed posterior distributions of the differences between them for each variance component. We calculated adjusted repeatability (R) separately for each predator condition. Repeatability was estimated as the proportion of total phenotypic variance attributable to among-individual differences: *R* = $\frac{V_{\mathrm{Among}}\;}{V_{\mathrm{Among}\;}+\;V_{\mathrm{Residual}}}$. The among-individual variance $\left(V_{\mathrm{Among}\;}\right)$ was extracted as the variance of the random intercepts for individual identity within each predator condition. Because our model is a DHGLM with fixed and random effects affecting the log of the residual standard deviation, we calculated the expected residual variance $\left(V_{\mathrm{Residual}}\right)$, representing within-individual variance accounting for among-individual variation in residual standard deviation. Specifically, for each predator condition, $V_{\mathrm{Residual}}$ was calculated by exponentiating the sum of: (i) the fixed effect intercept(s) for log(residual standard deviation), and (ii) half the among-individual variance in log(residual standard deviation), then squaring this quantity to convert from the standard deviation scale to the variance scale. This approach accounts for the fact that when log(*σ*) varies among individuals, the expected residual variance incorporates both the mean log(*σ*) and the variance in log(*σ*) among individuals. We calculated repeatability from the posterior distribution and estimated the difference in repeatability between predator conditions.

The model also included tadpole size, number of conspecifics in the pool of origin and experimental batch as fixed effects. We included tadpole size (scaled and mean-centered) to control for size differences; and the number of conspecifics also present in the tadpoles' pool of origin (scaled and mean centered) to control for density differences among the pools. The pool of origin was modeled as a random effect in both the mean and dispersion parts of the model to account for nonindependence among individuals originating from the same pool and control for unmeasured variation among the different pools.

Differences between predator-naïve and predator-exposed estimates are reported as significant if the 95% HPDI for the difference between them does not cross 0.

## Results

### Effect of predation at the group level

**Figure 2:**
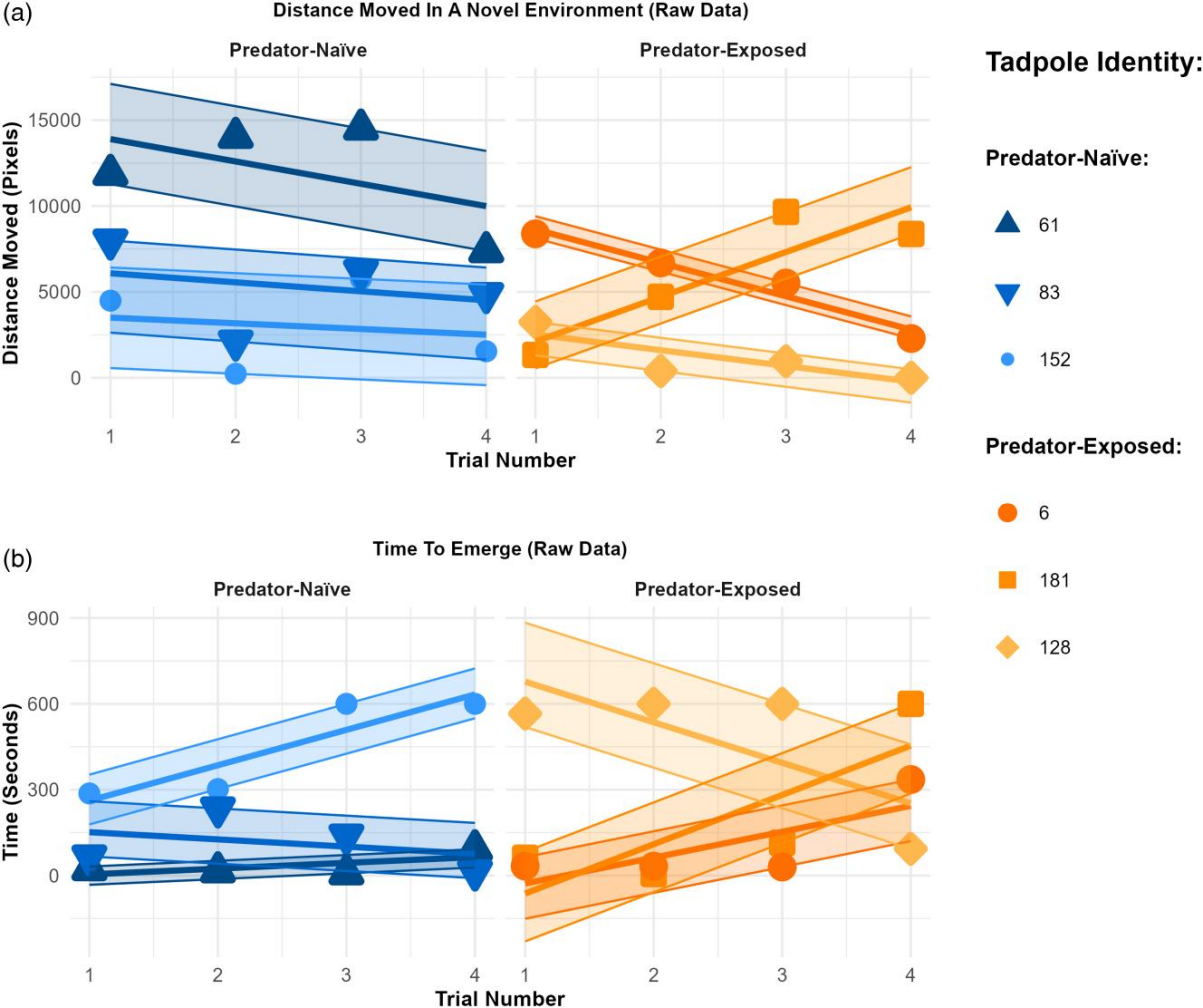
Raw data illustrating a) distance moved in a novel environment and b) time to emerge from a shelter for 6 individual tadpoles (three predator-exposed and three predator-naïve). For each individual, a linear model was fitted across trials to visualize behavioral trajectories (plasticity to repeated testing), and shaded ribbons represent the deviation of observed data points from these fitted slopes, illustrating within individual residual variation (predictability). Individuals were selected as those ranking within the top 10%, middle 10%, and bottom 10% for both time to emerge and distance moved, illustrating the range of behavioral strategies expressed within predator-exposed and predator-naïve groups.

**Figure 3:**
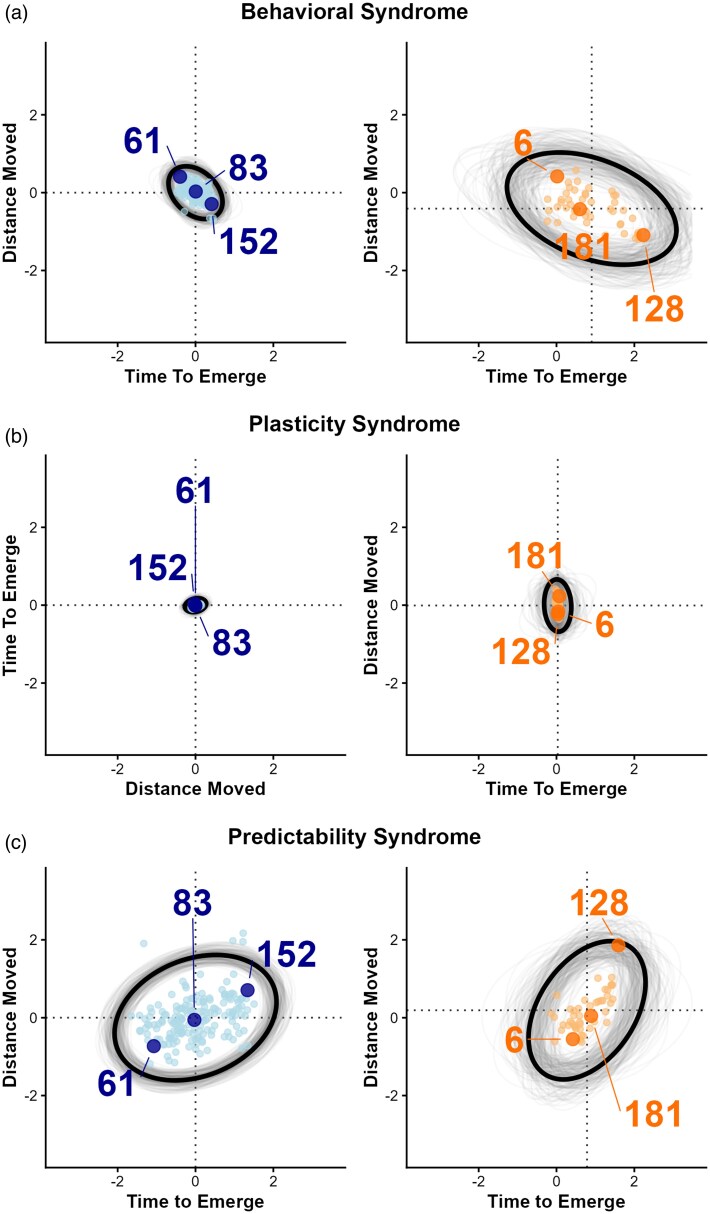
Ellipse plots showing the (co)variation between the posterior modes of individual-level random effects (BLUPs) from the DHGLM for the log-transformed distance moved in a novel environment (“Distance Moved”) and the time to emerge from a shelter (“Time to Emerge”) for a) initial behavior (intercept), b) behavioral plasticity across repeated testing (change from trials 1–4), and c) predictability (residual variation). The predator-naïve ellipse is centered on (0, 0), while the predator-exposed ellipse is shifted by the estimated predator effect from the model. Ellipse orientation indicates the correlation estimate, and ellipse size reflects the magnitude of among-individual variance. Bold ellipses represent posterior mode estimates, and grey ellipses illustrate uncertainty based on 300 randomly drawn samples from the posterior distribution. Highlighted individuals correspond to those identified in Fig. 2.

Predator-exposed individuals moved less in the novel environment (Figs. 2a and 3a) and took longer to emerge from a shelter on their first trial (Figs. 2b and 3a), than predator-naïve individuals (Table 1).

**Table 1:** Posterior estimates of phenotypic effects (group-level) for predator-exposed (“Exposed”) and predator-naïve (“Naïve”) individuals for 2 behaviors: distance moved in a novel environment (“Distance Moved”) and time to emerge from a shelter (“Time to Emerge”).

	Distance moved			Time to emerge		
	Mean	95% HPDI		Mean	95% HPDI	
		Lower	Upper		Lower	Upper
Effect of Predator Exposure at the Group Level: Initial Behavior						
Naïve	0.11	−0.14	0.33	**−0**.**34**	**−0**.**59**	**−0**.**10**
Exposed	−0.30	−0.85	0.28	0.55	−0.11	1.22
Δ Exposure	**−0**.**41**	**−0**.**79**	**−0**.**03**	**0**.**90**	**0**.**41**	**1**.**42**
Effect of Predator Exposure at the Group Level: Plasticity to Repeated Testing						
Naïve	−0.03	−0.07	0.01	**0**.**09**	**0**.**04**	**0**.**14**
Exposed	−0.04	−0.22	0.12	0.12	−0.07	0.36
Δ Exposure (Interaction between Predator Presence and Trial Number)	−0.01	−0.14	0.12	0.03	−0.13	0.22
Effect of Predator Exposure at the Group Level: Predictability						
Naïve	**−0**.**91**	**−1**.**11**	**−0**.**70**	**−0**.**59**	**−0**.**93**	**−0**.**23**
Exposed	**−0**.**72**	**−1**.**18**	**−0**.**29**	0.15	−0.30	0.61
Δ Exposure	0.19	−0.18	0.56	**0**.**79**	**0**.**29**	**1**.**30**

In both predator-exposed and predator-naïve groups, individuals did not show changes in distance moved in a novel environment with repeated testing at the population level (Table 1). However, in predator-naïve individuals, there was a significant increase in the time taken to emerge from the shelter with repeated testing (Table 1). For predator-exposed individuals, the 95% HPDI for the change in behavior across repeated tests crossed 0, indicating no significant change in time to emerge with repeated testing. Despite this, there was no significant difference in the effects of repeated testing between the predator-naïve and predator-exposed groups.

Predator-exposed and predator-naïve individuals did not significantly differ in the predictability of the distance they moved in a novel environment (Table 1; Figs. 2a and 3c). However, predator-exposed individuals were less predictable in their time to emerge from a shelter than predator-naïve individuals, as the predator-exposed individuals had significantly higher residual variation in their time to emerge than predator-naïve individuals (Table 1; Figs. 2b and 3c).

Estimates are reported for 2 behaviors: distance moved in a novel environment (Distance Moved) and time to emerge from a shelter (Time to Emerge). For each behavior, we show initial behavior (intercept), plasticity to repeated testing (slope), and predictability (residual variation). Predator-naïve individuals represent the model intercept, Δ Exposure shows the effect of predator exposure, and predator-exposed values are the sum of the intercept and Δ Exposure. Positive Δ Exposure indicates an increase due to predator exposure, while negative values indicate a decrease. Estimates are shown with their 95% HPDI; values for which the HPDI does not overlap 0 are highlighted in bold.

### Effect of predation on among-individual variation

Predator-exposed individuals showed greater levels of among-individual variation in their initial behavior in time to emerge from a shelter and in distance moved in a novel environment than predator-naïve individuals (Table 2; Fig. 3a). Predator-exposed individuals showed greater among-individual variation in how the distance they moved in a novel environment changed with repeated testing (plasticity) than predator-naïve individuals (Table 2; Figs. 2a and 3b). There were no differences in among-individual variation in plasticity in time to emerge from a shelter (Table 2; Figs. 2b and 3b). Among-individual variance in predictability was significantly greater than zero in both predator-exposed and predator-naïve individuals, with no difference between the 2 groups (Table 1; Figs. 2ab and 3c). Given that we found nonzero mean-variance correlation at the individual level (personality-predictability correlations within a trait in Table 2), we estimated how much of the variance in predictability was due to the variation in the mean (Supplementary S1).

**Table 2:** Posterior estimates for among-individual variance in initial behavior (intercept), behavioral plasticity across repeated testing (slope), and predictability (residual variation) for distance moved in a novel environment (“Distance Moved”) and time to emerge from a shelter (“Time to Emerge”) in predator-naïve (“Naïve”) and predator-exposed (“Exposed”) individuals.

	Distance moved			Time to emerge		
	Mean	95% HPDI		Mean	95% HPDI	
		Lower	Upper		Lower	Upper
Variance Among Individuals: Initial Behavior						
Naïve	**0**.**08**	**0**.**04**	**0**.**13**	**0**.**08**	**0**.**03**	**0**.**14**
Exposed	**0**.**33**	**0**.**13**	**0**.**66**	**0**.**75**	**0**.**27**	**1**.**62**
Δ Exposure	**0**.**27**	**0**.**02**	**0**.**55**	**0**.**71**	**0**.**12**	**1**.**42**
Variance Among Individuals: Plasticity to Repeated Testing						
Naïve	0.01	0.00	0.03	0.00	0.00	0.01
Exposed	**0**.**07**	**0**.**03**	**0**.**15**	0.01	0.00	0.10
Δ Exposure	**0**.**06**	**0**.**01**	**0**.**12**	0.02	−0.02	0.09
Variance Among Individuals: Predictability						
Naïve	**0**.**43**	**0**.**31**	**0**.**59**	**0**.**72**	**0**.**52**	**0**.**97**
Exposed	**0**.**51**	**0**.**25**	**0**.**96**	**0**.**36**	**0**.**13**	**0**.**77**
Δ Exposure	0.09	−0.25	0.53	−0.35	−0.73	0.05
Variance Within Individuals						
Naïve	**0**.**25**	**0**.**16**	**0**.**36**	**0**.**68**	**0**.**24**	**1**.**16**
Exposed	**0**.**42**	**0**.**19**	**0**.**74**	**2**.**34**	**0**.**90**	**4**.**27**
Δ Exposure	0.17	−0.10	0.50	**1**.**65**	**0**.**07**	**3**.**84**
Repeatability of Behavior						
Naïve	**0**.**24**	**0**.**12**	**0**.**35**	**0**.**12**	**0**.**02**	**0**.**21**
Exposed	**0**.**45**	**0**.**21**	**0**.**72**	**0**.**26**	**0**.**07**	**0**.**47**
Δ Exposure	0.21	−0.08	0.49	0.15	−0.11	0.36

For time to emerge, within-individual variance was significantly greater in predator-exposed individuals (Table 2). However, for distance moved in a novel environment, within-individual variance did not differ significantly between the predator conditions (Table 2). Both predator-naïve and predator-exposed individuals both showed repeatable behavior (Table 2). In both behaviors, predator-exposed and predator-naïve individuals did not significantly differ from one another in their repeatability (Table 2).

Estimates for among-individual variance are derived from the random effect standard deviations for the random effect of individual identity, squared to convert to variance. “Repeatability of Behavior” and “Variance within Individuals” estimates were calculated as described in the main text. Posterior estimates of the differences between predator-exposed and predator-naïve individuals (Δ Exposure) are also provided. Estimates are shown with their 95% HPDI; values for which the HPDI does not overlap 0 are highlighted in bold. For variance parameters, which cannot be negative, estimates are bolded if the lower HPDI exceeded 0.01.

### Effect of predation on behavioral correlations

For among-trait correlations, in predator-naïve individuals, we found a behavioral syndrome between distance moved in a novel environment and time to emerge from a shelter: individuals that traveled further in the novel environment emerged sooner from the shelter (negative correlation between intercepts; Table 3; Fig. 3a). In predator-exposed individuals, the correlation was in the same direction but the 95% HPDI overlapped zero, providing weaker evidence for a syndrome. The correlations were not different between predator-exposed and predator-naïve groups. There was no correlation between plasticity to repeated testing in either behavior in the predator-exposed or predator-naïve individuals, therefore there was no evidence for a plasticity syndrome (Table 3; Fig. 3b). There was a predictability syndrome in the predator-naïve group where individuals who were more predictable in their distance moved in a novel environment were also more predictable in the time taken to emerge (Table 3; Fig. 3c). Similarly, to the results for the behavioral syndrome, the 95% HPDI for the predator-exposed group did overlap zero, but the correlations were not different across predator-exposed and naïve groups. Overall, there was no difference between the predator-exposed and predator-naïve groups in behavioral syndrome, plasticity syndrome, or predictability syndrome (Table 3; Fig. 3a–c).

**Table 3:** Posterior correlation estimates at the among-individual level (personality, plasticity, and predictability correlations) for predator-naïve (‘Naïve’) and predator-exposed (‘Exposed’) tadpole groups, as well as the difference between predator exposure groups.

Correlation parameter	Naïve			Exposed			Δ Exposed—naïve		
	Mean	95% HPDI		Mean	95% HPDI		Mean	95% HPDI	
		Lower	Upper		Lower	Upper		Lower	Upper
Behavioral syndrome	**−0**.**36**	**−0**.**66**	**−0**.**03**	−0.38	−0.73	0.05	−0.03	−0.50	0.53
Plasticity syndrome	0.23	−0.37	0.72	−0.07	−0.72	0.64	−0.30	−1.16	0.61
Predictability Syndrome	**0**.**25**	**0**.**05**	**0**.**44**	0.42	−0.01	0.77	0.17	−0.26	0.60
Personality vs Plasticity									
DM Personality DM Plasticity	−0.30	−0.62	0.22	**−0**.**49**	**−0**.**80**	**−0**.**02**	−0.18	−0.76	0.39
TE Personality TE Plasticity	0.03	−0.53	0.63	0.02	−0.67	0.72	−0.00	−0.90	0.93
DM Personality TE Plasticity	−0.06	−0.56	0.52	−0.03	−0.69	0.66	0.03	−0.82	0.91
TE Personality DM Plasticity	−0.29	−0.66	0.09	−0.33	−0.73	0.16	−0.04	−0.62	0.57
Personality vs Predictability									
DM Personality DM Predictability	**−0**.**77**	**−0**.**95**	**−0**.**50**	**−0**.**54**	**−0**.**86**	**−0**.**07**	0.23	−0.18	0.72
TE Personality TE Predictability	**0**.**88**	**0**.**73**	**0**.**98**	**0**.**77**	**0**.**41**	**0**.**95**	−0.12	−0.47	0.15
DM Personality TE Predictability	**−0**.**31**	**−0**.**56**	**−0**.**06**	−0.17	−0.58	0.30	0.14	−0.31	0.70
TE Personality DM Predictability	**0**.**31**	**0**.**04**	**0**.**54**	**0**.**47**	**0**.**04**	**0**.**79**	0.16	−0.32	0.60
Plasticity vs Predictability									
DM Plasticity DM Predictability	−0.16	−0.54	0.23	−0.12	−0.60	0.38	0.04	−0.58	0.65
TE Plasticity TE Predictability	0.37	−0.20	0.81	0.16	−0.58	0.79	−0.22	−1.14	0.56
DM Plasticity TE Predictability	−0.17	−0.50	0.17	**−0**.**43**	**−0**.**79**	**−0**.**05**	−0.25	−0.77	0.30
TE Plasticity DM Predictability	−0.07	−0.57	0.42	−0.01	−0.69	0.66	0.06	−0.77	0.89

For within-behavior correlations, in distance moved in a novel environment and time to emerge, there were personality-predictability associations in both predator-exposed and predator-naïve individuals (Table 3). Individuals that moved more in a novel environment on their first trial were more predictable in their behavior, while individuals that took longer to emerge from a shelter were less predictable in their behavior. In the predator-exposed group, there was a personality-plasticity association in the novel environment assay, where individuals that initially moved further in a novel environment changed their behavior less with repeated testing. There was no evidence for a personality-plasticity association in the predator-naïve group, or in either group in the time taken to emerge from a shelter (Table 3). Despite the personality-plasticity association in the novel environment assay being present in the predator-exposed group and not the predator-naïve group, there was no evidence for a difference in correlations between the 2 groups (Table 3). There were no plasticity-predictability associations within distance moved in a novel environment or time to emerge (Table 3). There was no evidence for differences in personality-plasticity, personality-predictability or plasticity-predictability associations between predator-exposed and predator-naïve individuals.

Names of the parameters starting with “DM” refer to distance moved in a novel environment and “TE” refers to time taken to emerge from a shelter. Behavioral syndrome represents the correlation between individual intercepts (initial behavior) across the 2 behaviors; plasticity syndrome represents the correlation between individual slopes to trial (plasticity to repeated testing) across the 2 behaviors; predictability syndrome represents the correlation between individual residual variance (predictability) across the 2 behaviors. Posterior estimates for the difference between predator-exposed and predator-naïve groups are provided across all correlations (Exposed minus naïve). Estimates are displayed alongside their 95% HPDI; estimates in which the HPDI does not overlap 0 have been highlighted in bold.

### Other factors

Larger tadpoles moved further in a novel environment, but size had no impact on time taken to emerge from a shelter (Table 4). There was no effect of experimental batch on time to emerge (Table 4), but there was an effect of experimental batch on distance moved in a novel environment, with individuals moving more in experimental batch 2 and 5 than in batch 1 (Table 4). The density of tadpoles in an individual pool of origin affected neither the distance moved in a novel environment or the time taken to emerge from a shelter (Table 4) and pool of origin only explained a very small amount of variation in distance moved in a novel environment and time to emerge from a shelter at both the mean and residual level, with all of the 95% HPDIs including 0 (Table 4). There were no significant correlations at the pool level (Supplementary S2).

**Table 4:** Posterior estimates for the effect of size, conspecific number, experimental batch and pool of origin on 2 behaviors: distance moved in a novel environment and time to emerge from a shelter.

	Distance moved			Time to emerge		
	Mean	95% HPDI		Mean	95% HPDI	
		Lower	Upper		Lower	Upper
Intercept						
	0.11	−0.14	0.33	**−0**.**34**	**−0**.**59**	**−0**.**10**
Fixed Effects:						
Size	**0**.**09**	**0**.**04**	**0**.**14**	−0.05	−0.11	0.00
Experimental Batch 2	**0**.**18**	**0**.**04**	**0**.**31**	−0.09	−0.24	0.07
Experimental Batch 3	0.01	−0.14	0.17	−0.02	−0.20	0.16
Experimental Batch 4	−0.09	−0.28	0.10	0.18	−0.02	0.39
Experimental Batch 5	**0**.**30**	**0**.**08**	**0**.**52**	−0.12	−0.33	0.11
Conspecific Number	0.03	−0.07	0.14	−0.03	−0.12	0.07
Random Effects (Mean Model):						
Pool of Origin	0.02	0.00	0.13	0.01	0.00	0.13
Random Effects (Residual Model)						
Pool of Origin	0.01	0.00	0.13	0.07	0.00	0.35

Estimates are displayed alongside their HPDI at 95%. Estimates with HPDI which do not overlap zero have been highlighted in bold. Because variance parameters cannot be negative, estimates for random effects were bolded if their lower HPDI was above 0.01.

## Discussion

Predator exposure during development significantly influenced the behavior of *Allobates femoralis* tadpoles in a free-ranging population. Predator-exposed tadpoles exhibited reduced movement in novel environments and longer emergence times from the shelter on their first trial, and showed greater among-individual variation in initial behavior across both behaviors and in plasticity to repeated testing for distance moved. In predator-naïve individuals, a behavioral syndrome was present, with individuals that moved more in a novel environment emerging sooner from the shelter, and a predictability syndrome was also evident, with more predictable individuals in distance moved also being more predictable in emergence time; correlations were in the same direction in predator-exposed individuals but with weaker evidence. Personality-predictability associations were evident in both groups: individuals that moved more were more predictable in distance moved, while individuals that took longer to emerge were less predictable in emergence time. Only predator-exposed tadpoles demonstrated a personality-plasticity association in distance moved: individuals that initially swam further showed less change across repeated trials than those that initially moved less. Notably, behavioral correlations did not differ significantly between groups, suggesting that while developmental predator exposure shaped the magnitude of behavioral variation, it left the underlying correlation structure largely intact.

### Effect of predation at the group level

Tadpoles that developed in the presence of predators moved less in a novel environment and took longer to emerge from a shelter on their first trial. This result may be explained by phenotypic plasticity, whereby individuals adjust their behavior in response to predator cues. Similar patterns have been observed in other species, where prey reduce their activity levels when raised alongside predators (Scherer et al. 2024). In particular, this aligns with previous findings in larval anurans, which show reduced movement when they developed in the presence of predators (Anholt and Werner 1995; Relyea 2001; Van Buskirk 2001; Dijk et al. 2016; Gazzola et al. 2017; Hossie et al. 2017; Urszán et al. 2018; Castellano and Friard 2021), and spend more time hiding (Van Buskirk 2001; Gazzola et al. 2017). Similarly, larval newts have been found to spend more time concealed in leaf litter when raised with predators (Van Buskirk and Schmidt 2000). Alternatively, or additionally, these patterns could reflect selective removal, whereby more active individuals, those that move more or emerge faster from shelter, are preferentially preyed upon. For example, in the common frog (*Rana temporaria*), less active individuals were captured less frequently by dragonfly larvae predators (Dijk et al. 2016).

Contrary to our predictions, predator exposure did not significantly affect plasticity in emergence time across repeated trials. While predator-naïve tadpoles showed significantly longer emergence times with repeated testing, predator-exposed tadpoles exhibited a similar nonsignificant trend. The lack of significance is potentially due to reduced statistical power from smaller sample sizes of predator-exposed tadpoles. We had predicted that habituation rates would decrease under higher predation risk due to greater costs of erroneous habituation (Brown et al. 2013; Mitchell et al. 2024). Behavioral change across repeated testing may involve multiple concurrent processes, such as responses to time in the isolation tube, ontogenetic shifts, or their interactions, beyond habituation alone. If tadpoles integrate and respond to multiple environmental cues simultaneously, predictions based solely on habituation theory may be insufficient. This could also explain the lack of change in distance moved across trials: some individuals may increase while others decrease movement, either due to individual differences in responses to repeated testing, the balancing of opposing responses to different cues, or a lack of habituation to the novel environment assay. The absence of an effect of predator risk on plasticity aligns with (Martin and Réale 2008), who found no habituation differences in chipmunks across environments that differ in predation pressure, but contrasts with (Mitchell et al. 2024), who observed faster habituation in predator-exposed (compared with predator-naïve) guppies. Such discrepancies may reflect different testing set-ups, species-specific antipredator strategies, or differences in predator regime stability, which can alter the costs and benefits of behavioral plasticity.

We found that predator-exposed individuals were less predictable than predator-naïve individuals in their time to emerge, although predictability in distance moved in a novel environment did not differ between predator-exposed and predator-naïve individuals. This reduced predictability in time to emerge is consistent with findings in hermit crabs (*Pagurus bernhardus*) (Briffa 2013) and Italian tree frog tadpoles (*Hyla intermedia*) (Castellano and Friard 2021). However, contrasting evidence comes from agile frog tadpoles (*Rana dalmatina*), where predator presence increased predictability in activity levels (Urszán et al. 2018). Our findings support the idea that less predictable behavior may be an adaptive antipredator tactic, making individuals harder for predators to capture (Domenici et al. 2009; Briffa 2013). This result also reinforces that behavioral predictability is a meaningful trait and an important component of antipredator responses, rather than simply measurement error (Westneat et al. 2015).

### Effect of predation on among-individual variation

Predator-exposed individuals showed greater among-individual variation in distance moved and emergence time compared with predator-naïve individuals. This pattern appears across species (Toscano et al. 2014; Ehlman et al. 2019; Tariel et al. 2020; Mitchell et al. 2024) but contrasts with results from Amazonian mollies (*Poecilia formosa*), where predator presence reduced variation (Scherer et al. 2024). Increased variation may result from gene-environment interactions, as genetically diverse *A. femoralis* individuals may respond consistently but differently to predator exposure. This pattern is supported by increased among-individual variation under higher predation risk in other studies on genetically diverse individuals (eg Toscano et al. 2014; Urszán et al. 2015; Ehlman et al. 2019; Tariel et al. 2020; Mitchell et al. 2024). Alternatively, variable individual predator experience could explain this pattern. Although isolation tubes limited direct contact during testing, individuals likely experienced different interaction levels before tube placement, and predators remained visible and could interact differentially with different tubes. Consequently, some individuals may have had minimal exposure while others experienced close encounters, creating variability in behavioral responses. Scherer et al. (2024) controlled for both genetic background (by using clonal fish) and predator experience and found reduced among-individual variation in predator-exposed individuals. Together, this supports that increased among-individual variation seen under predation risk elsewhere may be driven by differences in individual experience and gene-environment interactions.

Predator-exposed individuals showed greater among-individual variation in plasticity to repeated testing for distance moved, although not for emergence time. The greater among-individual variation in plasticity parallels Urszán et al. (2018), who found that *R. dalmatina* tadpoles exposed to predator cues during development showed increased among-individual variation in plasticity. While our study examined plasticity through repeated testing and Urszán et al. (2018) examined plasticity in response to predator cue presence, both suggest that predation exposure increases among-individual variation in plasticity. This increased variation could arise from genetic differences in how individuals respond to predators or from varying levels of predator experience during development. We did not find evidence that predator exposure affected among-individual variation in predictability.

Changes in behavioral repeatability can arise through different mechanisms; either increased among-individual variation or decreased within-individual variation. In our study, repeatability was numerically higher under predator exposure for both behaviors, although these differences were not statistically significant. For distance moved in a novel environment, repeatability increased from 0.23 (95% HPDI: 0.12 to 0.35) in predator-naïve individuals to 0.45 (95% HPDI: 0.21 to 0.72) in predator-exposed individuals, driven by increased among-individual variation, while within-individual variation did not differ significantly. The lack of statistical significance, despite increased among-individual differences, may reflect limited statistical power (as indicated by the wide HPDI for predator-exposed individuals) or a small, nonsignificant increase in within-individual variation. For time to emerge, repeatability increased from 0.12 (95% HPDI: 0.02 to 0.21) in predator-naïve individuals to 0.26 (95% HPDI: 0.07 to 0.47) in predator-exposed individuals. In this case, both among- and within-individual variation increased, which could explain the absence of a significant difference. Previous studies have reported mixed results: some report higher behavioral repeatability following predator exposure in mosquitofish (*Gambusia affinis*) (Ehlman et al. 2019), crabs (*Panopeus herbstii*) (Toscano et al. 2014), and tadpoles of both the moor frog *Rana arvalis* (Płaskonka et al. 2024) and the agile frog (*R. dalmatina*) (Urszán et al. 2015). In contrast, predator-naïve Italian treefrog tadpoles (*H. intermedia*) exhibited higher repeatability than predator-exposed individuals (Castellano and Friard 2021). We need to decompose repeatability into its variance components to determine whether studies are comparing equivalent patterns. For instance, Castellano and Friard (2021) found reduced repeatability in predator-exposed tadpoles as individuals became less predictable under predation. We also observed that predator-exposed individuals were less predictable in their time to emerge; however, because among-individual variation also increased, overall repeatability did not decrease. Only by partitioning repeatability into its among- and within-individual components can we identify the mechanisms driving consistent individual differences in behavior.

### Effect of predation on behavioral correlations

There was evidence of a behavioral syndrome in predator-naïve individuals: those that initially moved more in a novel environment also emerged sooner from the shelter. Predator-exposed individuals showed the same negative correlation, though with weaker evidence, possibly due to smaller sample size. Importantly, these correlations did not differ significantly between predator-exposed and predator-naïve groups. This contrasts with previous studies on tadpoles (Urszán et al. 2015), sticklebacks (Bell and Sih 2007; Dingemanse et al. 2007), and mosquitofish (Ehlman et al. 2019) that found behavioral syndromes only in, or strengthened by, predator presence. This difference may reflect distinct evolutionary histories. Dingemanse et al. (2007) found behavioral syndromes only in sticklebacks that co-occur in ponds with predators. This could be explained by limited gene flow between ponds, and the syndrome only evolving where predators were present. Similarly, Bell and Sih (2007) found that behavioral syndromes emerged only after predator introduction in populations without a history of strong predation. If behavioral syndromes evolve specifically in response to predation pressure, they would not be expected in these populations prior to predator introduction, as they lack such selective conditions. In contrast, our study population has likely experienced sustained high predation pressure (predators occupied roughly half of available pools during this study). Additionally, substantial gene flow occurs between pools, as male frogs often distribute tadpoles from the same clutch across multiple pools (Erich et al. 2015). Therefore, even if behavioral syndromes evolved in response to predator-driven selection, they would be present across all pools due to gene flow, which could explain why we observed no difference between behavioral syndromes in predator-exposed and predator-naïve groups.

A predictability syndrome was also present in predator-naïve individuals, with those that were more predictable in their distance moved in a novel environment were also more predictable in their time to emerge from a shelter. Predator-exposed individuals showed a similar trend, albeit with greater uncertainty around the estimate, consistent with reduced sample size, and the correlation did not differ significantly between predator conditions. This suggests a shared basis for predictability across these behaviors, potentially arising from correlated selective pressures or genetic correlations (O’Dea et al. 2022). This relationship did not differ between predator-exposed and predator-naïve individuals, indicating that the predictability syndrome is a stable feature of the population and unaffected by developmental exposure to predators. In contrast, we found no evidence for a plasticity syndrome. Individuals that showed greater plasticity in distance moved in a novel environment (changing distance more with repeated testing) did not necessarily show greater plasticity in emergence time. However, we observed generally low among-individual variation in plasticity to repeated testing, which limits the statistical power to detect correlations between traits. This low variation could reflect genuinely low variation in this trait with the population or insufficient assays trials, though variation in plasticity to repeated testing has been detected with comparable trial numbers (Dingemanse et al. 2012).

For within-behavior correlations, there was a personality-predictability association in both behaviors. Individuals that initially moved more in the novel environment were more predictable, and individuals that initially took less time to emerge from a shelter were also more predictable. This suggests that in both traits, “bolder” individuals (in this case, individuals exhibiting higher levels of behaviors that are typically downregulated under predation) were more predictable. This result contrasts with the expected pattern; wherein bolder personalities could benefit from less predictable behavior as an antipredator strategy (Highcock and Carter 2014). Additionally, only in the predator-exposed group did we find a personality–plasticity association for distance moved in a novel environment, where individuals that traveled farther initially showed less change in movement across repeated trials. This plasticity-personality association was likely only present in predator-exposed individuals as significant among-individual variation in plasticity was only present in distance moved in a novel environment among predator-exposed individuals. However, the correlation did not differ significantly between predator-exposed and predator-naïve groups. The predator-specific detection likely reflects greater among-individual variation in plasticity for distance moved in predator-exposed individuals, potentially arising from gene–environment interactions or differential developmental experiences. Such variation enables the detection of correlations between personality and plasticity, as these can only be observed when sufficient variation exists in both traits. All our results for within-behavior correlations points to a similar pattern: less within-individual variation in “bolder” individuals. Such individuals may show reduced within-individual variation as they may be closer to a hypothetical “ceiling” of behavior, where any fluctuation beyond a certain threshold could prove detrimental or fatal (Stamps and Groothuis 2010; O’Dea et al. 2022).

### Selective removal versus plastic response

The effects of predator presence on behavior could arise from selective removal of certain behavioral types or from plastic behavioral changes in response to predation risk. Plasticity would shift both the mean and range of behavior as individuals adjust beyond the original variation, while selective removal would shift the mean but not the range, since it removes behavioral types without extending beyond initial population variation. However, ceiling and floor effects in our measures prevent us from detecting range changes. Time to emerge has an upper limit at 600 s (when the assay ended), while distance moved in a novel environment has a lower limit at 0 (no movement). The impact of predation pushes the time to emerge closer to the ceiling effect and distance moved in a novel environment closer to the floor effect. Since some individuals in both predator-exposed and predator-naïve reached these limits, we cannot determine whether predator-exposed individuals' behavior extends beyond the range observed in predator-naïve individuals. This prevents us from distinguishing between selective removal and plasticity as mechanisms.

A third possible mechanism is genetic variation between tadpoles in pools with and without predators, which could contribute to the observed differences alongside selection and plasticity. *A. femoralis* males distribute clutches across multiple pools (Erich et al. 2015; Ringler et al. 2018), therefore substantial genetic overlap among pools likely exists. However, some genetic differentiation could occur if males systematically differ in their deposition patterns. Anurans generally prefer depositing their young in pools without predators (Buxton and Sperry 2017), including in this *A. femoralis* population where tadpole deposition likelihood decreased with increasing numbers of dragonfly larvae (Erich et al. 2015). Additionally, males had more offspring surviving to adulthood if they were exploratory (in combination with other traits), perhaps due to enhanced ability to find better quality pools without predators (Peignier et al. 2023). If such behavioral differences between males are heritable, as found in great tits (Drent et al. 2003) and burrowing owls (Carrete et al. 2016), this could create behavioral differences between tadpoles in different pool types. To disentangle whether behavioral differences arise from plastic responses to predation cues, selective removal, or genetic differentiation between pool types, future work should compare individuals of known genotypes in common garden experiments using nonconsumptive predator cues versus controls.

## Conclusion

Overall, early-life predation risk had strong effects on both among- and within-individual variation in tadpole behavior, though behavioral correlations remained largely similar between predator-naïve and predator-exposed individuals. Increased among-individual variation in predator-exposed populations may provide greater phenotypic diversity for selection to act upon. However, the persistence of a stable correlation structure among behaviors may constrain the independent evolution of traits, potentially limiting adaptive responses when different behavioral combinations are favored in the presence and absence of predators. These results suggest that early environmental experiences can shape patterns of individual behavioral variation, providing insight into how “animal personality” develops in nature.

## Supplementary Material

arag041_Supplementary_Data

## Data Availability

Analyses reported in this article can be reproduced using the data provided by McPherson et al. (2026). The code for the python tracking script can be accessed via GitHub (josiemcpherson/AllobatesFemoralisTadpolePredator).
