# Supplementary material for: Predation risk shapes among- and within-individual variation in behavior in poison frog tadpoles
Source: Behav Ecol. 2026 Apr 20;37(4):arag041. doi: 10.1093/beheco/arag041 (PMC13190278; doi:10.1093/beheco/arag041)
Supplement: arag041_Supplementary_Data [file arag041_supplementary_data.docx]

**Supplementary Materials:**

**1:**

There was significant among-individual variation in predictability in both groups, but predator-naïve and predator-exposed individuals did not differ in the magnitude of among-individual differences in predictability in either behavior (Table 1; Figure 3c). To determine whether among-individual differences in predictability were due only to mean-variance relationships rather than genuine differences in predictability, we examined the covariance between individual intercepts for initial behavior and individual intercepts for residual standard deviation. We calculated the adjusted variance in predictability using the formula V[1,1] - (V[1,2]²)/V[2,2], where V[1,1] is the among-individual variance in log(sigma) intercepts (i.e., how much individuals differ in their behavioral predictability), V[2,2] is the among-individual variance in individual intercepts (i.e., how much individuals differ in their initial behavior), and V[1,2] is the covariance between these individual intercepts. This removes the component of variance in individual predictability that can be explained by mean-variance scaling. Mean-variance relationships explained substantial portions of the apparent among-individual differences in predictability. For distance moved in a novel environment, mean-variance relationships explained 58% of individual variance in predictability in predator-naïve tadpoles and 28% in predator-exposed tadpoles. For time to emerge, these relationships explained 76% and 55%, respectively.

**2:** Among-individual correlations at the pool level from the multivariate distributional model. Personality refers to differences in behavioural intercepts; predictability refers to residual standard deviation. DM = distance moved; TE = time to emerge. Values are posterior means with 95% highest posterior density intervals (HPDI).

|  | 95% HPDI | | |
| --- | --- | --- | --- |
|  | Mean | Lower | Upper |
| DM Personality TE Personality | -0.18 | -0.87 | 0.70 |
| DM Personality DM Predictability | -0.16 | -0.87 | 0.74 |
| TE Personality TE Predictability | 0.28 | -0.65 | 0.90 |
| DM Predictability TE Predictability | 0.18 | -0.71 | 0.87 |
| DM Personality TE Predictability | -0.19 | -0.87 | 0.66 |
| TE Personality DM Predictability | 0.11 | -0.74 | 0.86 |
